# The Differences Between Childhood and Adult Onset Antiphospholipid Syndrome

**DOI:** 10.3389/fped.2018.00362

**Published:** 2018-11-27

**Authors:** Chris Wincup, Yiannis Ioannou

**Affiliations:** ^1^Department of Rheumatology, University College London, London, United Kingdom; ^2^Arthritis Research UK Centre for Adolescent Rheumatology, University College London, London, United Kingdom

**Keywords:** antiphospholipid syndrome, vascular thrombosis, pregnancy morbidity, pediatrics, anti-coagulation

## Abstract

Antiphospholipid syndrome (APS) is a rare autoimmune disease of unknown etiology that represents a leading cause of acquired thromboembolism and recurrent miscarriage. It is characterized by the persistent elevated presence of pathogenic antiphospholipid auto-antibodies directed against cardiolipin, ß2-glycoprotein-I, and/or a positive lupus anticoagulant test. As with many autoimmune disorders, the pathogenesis of APS is believed to be the result of a complex interaction between environmental triggers and genetic predisposition. Although more common in adults, APS occasionally manifests in the neonatal period and throughout childhood. Adut-onset APS classification criteria are poorly validated to the pediatric population (in which pregnancy related complications are seldom seen) and as a result, assessment of the prevalence of the disease in childhood is difficult. Thromboembolic events seen in children include deep venous thrombosis in addition to stroke and pulmonary embolism, which can lead to significant long-term disability. The disease can be classified as either primary (when occurring in isolation) or secondary, in which the disease is diagnosed in the context of another underlying disease, most commonly systemic lupus erythematosus. A variety of laboratory and clinical difference are seen between pediatric and adult-onset APS. The marked female predominance seen in adult-onset disease is less evident in childhood where the gender split is more evenly spread. In addition, children with APS are at a higher risk of recurrent thromboembolism than adults. The treatment of childhood-onset APS is challenging due to a lack of large-scale prospective studies in the pediatric population. Therapeutic options are often based upon treatment guidelines that have been based upon literature from the adult-onset form of the disease. In the majority of cases, treatment is focused on the prevention of further thrombosis through treatment with long-term anti-coagulation therapy. The evidence for the use of antiplatelet agents (such as aspirin) and hydroxychloroquine is inconclusive. It is important to remember that anti-coagulation can have significant lifestyle implications for the child with APS and it is essential to consider potential implications relating to school and recreational activities, with contact sports often discouraged due to the increased risk of bleeding.

## Introduction

Antiphospholipid syndrome (APS) is a rare autoimmune disorder characterized by recurrent pregnancy morbidity (PM) and vascular thrombosis (VT). It occurs in the presence of persistent pathogenic antiphospholipid antibodies (aPL) (1). These are a heterogenous group of antibodies directed against glycoproteins (2). Standard testing of these antibodies in clinical practice include detecting those directed against cardiolipin (aCL) and/or ß2-glycoprotein-1 (ß2GPI) by enzyme-linked immunosorbent assay (ELISA). In addition, a functional coagulation assay is used to test for the presence of the circulating lupus anticoagulant (LAC) (3). These antibodies must be persistent, with classification criteria emphasizing the importance of positive tests being repeated at an interval of no >12 weeks (4). It is essential that a positive test is repeated as a number of alternate conditions may result in a false positive detection of aPL antibodies. This includes a variety of infections, most classically syphilis (5). The diagnosis of APS may be complicated further by the fact that no one single assay is able to identify all pathogenic antibodies related to the disease (6).

The most common presentations of the disease are often the result of PM and VT in adults. With regards to pregnancy related symptoms, these more frequently present with recurrent fetal loss. Less commonly the disease may be associated with pre-eclampsia and pre-term delivery (7, 8). Vascular thrombotic manifestations include both venous and arterial systems such as deep vein thrombosis (DVT), pulmonary embolism (PE), stroke, transient ischemic attack (TIA), and myocardial infarction (MI) amongst the most frequently seen events in adults (9, 10). Classical cutaneous manifestations including livedo reticularis has long been associated with APS. Patients may develop neurological symptoms including chorea and epilepsy. Other than VT, other hematological manifestations such as thrombocytopenia and hemolytic anemia are also commonly seen in association with the disease (11, 12).

Although there are a number of features that are in common with adult onset disease, there are numerous key differences that are unique in the case of children who develop APS. PM is often not applicable to the pediatric population and the risk of VT is similarly lower than in adult populations, thus making the diagnosis of APS in children challenging (13, 14). Furthermore, decisions relating to anti-coagulant treatment in young patients with the disease requires careful consideration and have potentially significant effects on lifestyle of the child and their family. In the context of pediatric-onset APS, there is a lack of evidence relating to both treatment and long-term outcomes with consensus drawn from a number of small studies and case reports. Recommendations for treatment are often derived from the study of the disease in larger adult populations (14).

## Pathogenesis of APS

Although the exact pathogenesis of APS is not fully understood, significant advances have been made in recent years with regards to gaining a greater understanding of the underlying mechanisms that drive the development of the disease. It is believed that a combination of abnormalities in endothelial cells, monocytes and complement play a key role the development of both PM and VT. In terms of the pathogenesis of APS, the “two hit” hypothesis is the most well described and widely accepted mechanism of thrombus formation. This pathogenic principle is supported by the fact that the clinical symptoms of VT and PM only occur occasionally despite the persistent presence of aPL antibodies. This importantly highlights that antibodies alone do not explain the whole clinical picture. The so called “first hit” occurs as a direct consequence of aPL antibodies, which results in activation of platelets, monocytes, and endothelial cells. Furthermore, aPL antibodies induce downregulation of naturally occurring anticoagulants and impair fibrinolysis (15–18). This lowers the threshold for developing a thrombotic event. The “second hit” relates to an additional factor that further exacerbates a pro-thrombotic state (for example inflammation), which in combination with aPL antibodies ultimately induces thrombosis (19). The theory of a “second hit” is further supported by recent studies, which have implicated Toll Like Receptors (TLR) in mediating the inflammatory response in the pathogenesis of APS (20, 21). However, in the context of pediatric APS, there is a notable absence of other classical pro-thrombotic risk factors including both pathological states (such as hypertension) and lifestyle related factors such as smoking. Therefore, this may suggest that another key risk factor is present in the children who develop the disease. Registry data suggests that the rates of APS associated with malignancy (another pro-thrombotic state) are significantly lower in patients with pediatric APS (<1% of cases) when compared with adults with the disease, which can be associated with solid organ malignancies (22, 23). This may similarly suggest that the so-called “second hit” in childhood is the result of an alternative mechanism or is acting via a different pro-thrombotic state than is seen in adults with the disease.

It is obvious that thrombus formation is central to the VT manifestations of APS but it should also be noted that this has been implicated in the PM related complications of the disease. The main mechanism associated with fetal loss is suggested to be due to the result of thrombus formation within the placental vasculature, which causes an impairment in maternal-fetal blood exchange (24). In addition, in many cases of PM related complications of APS, histological examination of the placenta has demonstrated the presence of thrombosis and infarction (25–27).

As with many autoimmune diseases, APS is believed to occur as a result of a complex interaction of environmental and genetic factors. It has been suggested that a loss immune tolerance is induced by bacterial or viral antigens that contain similar sequences to phospholipids and results in cross reactive T cells (28, 29). In terms of the underlying genetic basis of APS, a number of associations have been identified from studies in both familial and non-familial cases of the disease. In particular, human leukocyte antigen (HLA)-DR, and DQ haplotypes show the most consistent association with the disease (30, 31). A variety of loci have been implicated with a susceptibility for APS including HLA-DR4, DR7, DR9, DR13, DR53, DQ6, DQ7, and DQ8 major histocompatibility complex II (MHC II) alleles (32). Animal studies further support a genetic basis to the disease with MHC II identified as being vital for producing aPL after immunization with ß2GPI (33). Several non-MHC genes have been shown to increase the susceptibility for APS including IFR5 and STAT4 (34, 35).

## Diagnosis

The diagnosis of APS in childhood is often a challenge for clinicians as young patients display a number of key difference when compared with those who present with the disease in adulthood. Given the lack of large prospective studies of the disease in children it is difficult to estimate the prevalence of the disease in this group with any certainty. In 2004, the pediatric APS (Ped-APS) registry was established as a collaborative project between the European Forum of Antiphospholipid syndrome and the Juvenile Systemic Lupus Erythematosus Working Group of the Pediatric Rheumatology European Society (PReS). The aim was to undertake global multicentered controlled studies in a large group of young patients with APS. Results from the international registry reviewing characteristics from 121 patients with pediatric APS found the mean age of onset was 10.7 years, with APS reported in neonates and throughout the adolescent period (22). Cases of pediatric APS showed an even split between males and females (1:1.2) (36). In adult-onset disease, there is a female predominance quoted in the region of 1:5 (37). This is similar to the data on systemic lupus erythematosus (SLE), in which adult-onset disease shows a propensity to effect females (1:9 males to females), whereas in pediatric and juvenile-onset disease SLE the ratio is closer 1:5 and in the pre-adolescent population closer to 1:1 (38).

### Definitions and classification criteria

Pediatric APS is defined as the onset of APS in those under the age of 18 years old (39). In both pediatric and adult-onset APS, the disease can be defined as either primary or secondary APS. Secondary APS refers to the state in which the disease occurs in combination with another connective tissue disease (CTD). Secondary APS in children is most commonly associated with systemic lupus erythematosus (SLE) (37). The disease has also been described in children with various coexisting disorders including juvenile idiopathic arthritis (JIA), juvenile dermatomyositis, Henoch-Schönlein purpura, autoimmune thyroiditis, hemolytic-uremic syndrome, Behçet's disease, rheumatic fever and malignancy (22, 40–44).

In the absence of another disease the condition is defined as primary APS. Although poorly defined between 38 and 50% of cases of childhood-onset APS are said to be primary (22). In comparison, primary APS in adults is said to account for more than half of cases (45). The lower proportion of primary APS seen in children may due to the low incidence of PM seen in children, but may also be related to an ascertainment bias if children without a known autoimmune disease are less frequently investigated for aPL when presenting with a VT event. Furthermore, a number of cases of primary pediatric APS are later revised as secondary APS after a subsequent diagnosis of SLE is made (36, 46). The proportion of patients who progress from primary to secondary forms of the disease are said to be higher in those who develop the disease during childhood when compared with those who suffer from the adult form of the disease (46, 47).

The study of pediatric-onset APS is complicated by the fact that the diagnostic criteria used in adults is not reliably validated in children as a key feature relates to the occurrence of PM. The updated Sapporo criteria for adult-onset APS is defined in Table 1 (4). Currently there is no universally accepted validated diagnostic criteria for pediatric APS. This poses a variety of challenges in particular with regards to conducting research and clinical trials. In 2016, the Single Hub and Access point for pediatric Rheumatology in Europe (SHARE) initiative highlighted that whilst the adult criteria for APS were specific for pediatric-onset disease they lacked sensitivity. Recommendations from the group presented at the 15th International Congress on Antiphospholipid Antibodies suggested that new classification criteria are required for pediatric APS and these should incorporate both thrombotic and non-thrombotic manifestations of the disease (48).

**Table 1:** The 2006 update Sapporo Criteria for a diagnosis of antiphospholipid syndrome (APS) requires at least 1 of the following clinical criteria and 1 of the following laboratory criteria.

**Clinical criteria**	*Vascular thrombosis (VT)* One or more clinical episodes of arterial, venous, or small vessel thrombosis in any tissue or organ. Thrombosis must be confirmed by objective va lidated criteria. For histological confirmation, thrombosis should be present without significant evidence of inflammation in the vessel wall. This can be further subclassified as being in the presence or the absence of additional risk factors for thrombosis.
	*Pregnancy morbidity (PM)* One or more unexpla ined deaths of morphologically normal fetuses at or beyond the l0th week of gestation, with normal feta l morphology documented by ultrasound or by direct examination of the fetus; 1 or more premature births of morphologically normal neonates before the 34th week of gestation because of eclampsia or severe pre-eclampsia defined according to standard definitions or recognized features of placental insufficiency; 3 or more unexpla ined consecutive spontaneous abortions before the lOth week of gestation, with maternal anatomical or hormonal abnormalities and paternal and maternal chromosomal causes excluded.
**Laboratory criteria**	*Lupus anticoagulant* is present in the plasma *Anticardiolipin antibody* of IgG or IgM isotype in serum or plasma is present in medium or high titer (>40 IgG phospholip id [GPL] units or IgM phospholipid [MPL] units, or >99th percentile), by ELISA *Anti-beta2-glycoprotein I antibody* of IgG and *I* or IgM isotype in serum or plasma (in titer >90th percentile). by ELISA

*Laboratory criteria must be positive on 2 or more occasions,at least 12 weeks apart*.

### Clinical features

A number of small studies have found that the most common presenting feature of pediatric APS is venous thrombosis in particular those involving the lower limbs (predominantly in the form of deep vein thrombosis) (22, 49). Arterial and small vessel thrombosis are the next most commonly observed manifestation (22). The cerebral circulation is the most common site of arterial thrombosis, but it also occurs within the retinal, coronary, hepatic and mesenteric circulation (50). Complications of thrombosis, often in combination with Raynaud's, can also include digital ulceration. These are rarely seen in children and so should raise suspicion of APS in any young patient presenting with these symptoms. Recent data, including results from the Ped-APS registry, has indicated that patients with pediatric APS are at an increased risk of recurrent thrombosis when compared with the adult-onset form of the disease (22, 51). Whether this is related to a more severe pathogenic phenotype or issues related to treatment such as adherence remain poorly understood.

Cerebral ischemia may occur as a result of cranial arterial thrombosis resulting in stroke or TIA. Both of these are extremely rare in childhood and therefore a diagnosis of APS should be considered in all cases (52, 53). Interestingly, Ped-APS registry data suggests that the incidence of cerebrovascular events as a proportion of overall APS are said to be higher in pediatric APS than in adults with the disease (22). Non-thrombotic neurological symptoms can also be non-specific and include migraine and headache (54). A wide variety of additional features of central nervous system (CNS) related APS symptoms include epilepsy, chorea and transverse myelitis although unlike adults with APS, these have been reported as isolated case reports and are less common manifestations when compared to adult onset APS (14, 55, 56). In addition, thrombosis can cause a variety of ocular disorders including amaurosis fugax and ischemic retinopathy secondary central retinal artery/venous occlusion (57–59). Myocardial infarction (MI) may be the result of coronary thrombosis secondary to APS and carries a high risk of mortality (60, 61). In addition, valvular heart disease is another rare manifestation of the disease (62). In terms of pulmonary manifestations of pediatric APS, pulmonary thromboembolism is a frequent presenting symptom of the disease and should raise the clinical suspicion of the diagnosis of APS in any child in the absence of an alternate cause for thrombosis (63, 64). Pulmonary hypertension may result from chronic pulmonary vaso-occlusive disease and typically is associated with poor long term outcomes (49). Furthermore, pulmonary fibrosis has also been described in the context of pediatric APS (although the majority of cases are in those with coexisting SLE) (57). Thrombosis related renal manifestations of pediatric APS including thrombotic microangiopathy and in some cases this may result in end stage renal failure necessitating renal replacement therapy (65, 66). Cases of focal proliferative glomerulonephritis has also been described in pediatric primary APS (66, 67). Furthermore, bilateral adrenal infarction secondary to thrombosis has also been reported as a rare cause of primary adrenal insufficiency in children with primary APS (68).

In addition to thrombosis, it is now universally accepted that a wide array of systemic symptoms that are also frequently seen in adult-onset disease are similarly associated with the pediatric form of the disease. These more generalized symptoms include dermatological manifestations, which can range from classical livedo reticularis to the more rarely seen severe complication of purpura fulminans (69). Aside from thrombosis, various hematological disorders have been reported in the context of pediatric APS including hemolytic anemia, leukopenia, thrombocytopenia and cryoglobulinemia (70).

The various clinical manifestations of pediatric APS are summarized in Table 2.

**Table 2:** Clinical features associated with pediatric antiphospholipid syndrome.

**Neurological**	Transverse myelitis Migraine Chorea Cognitive impairment Seizures Stroke *I* trans ient ischemic attack Venous sinus thrombosis Ocular ischemia
**Hematological**	Venous thrombosis Arterial thrombosis Thrombocytopenia Leukopenia Hemolytic anemia
**Dermatological**	Livedo reticularis Raynaud 's phenomenon Purpura fu lminans
**Cardiovascular**	Myocardial infarction Cardiac valvular disease
**Pulmonary**	Pulmonary embolism Pulmonary hypertension Interstitial fibrosis
**Renal**	Antiphospholipid nephropathy Renal thrombotic microangiopathy
**Endocrine**	Primary adrenal insufficiency (secondary to adrenal infarction)

### Serological abnormalities in APS

As outlined in Table 1, the diagnosis of APS occurs in the context of persistently positive aPL antibodies. Pediatric-onset APS demonstrates a variety of key serological differences when compared with adult-onset disease. The most commonly occurring aPL antibody in pediatric APS is directed against cardiolipin, which has been quoted as being present in approximately 80% of cases. In comparison, this has been reported to be positive in only just over half of adult cases of APS. In addition, LAC is seen in approximately 70-90% of pediatric patients with APS and by comparison is present in half of adult cases (22, 71). The presence of a persistently positive anti-ß2GPI antibody was found to be similar in both adult and pediatric forms of the disease. Multiple positivity is also a common finding in pediatric APS (71). A study by Avčin et al., in which auto-antibody titers were measured in 61 cases of pediatric APS suggested that the normal ranges for these antibodies are different from those used in adult cases. Furthermore, a high proportion of transient aPL antibody positivity was seen in children without the symptoms of APS and this was felt to be likely related to previous exposure to infection (72).

In recent years the role of antibodies to the amino-terminal domain of ß2GPI, domain 1 (DI), have been studied in detail in adults with APS. This has been shown to represent the pathogenic sub-population of anti- ß2GPI antibodies, directed against a conformational epitope present in the first domain of ß2GPI. Studies have identified that anti-DI antibodies are present in patients with APS and are associated VT manifestations of the disease (73). There is also evidence to suggest that anti-DI antibodies may be present in the absence of other aPL antibodies in cases of so called “seronegative APS” (74). Currently there are however no studies of the prevalence of anti-DI antibodies in childhood onset APS.

In the context of pediatric secondary APS, it is important to screen for other auto-antibodies that may be indicative an additional autoimmune process such as SLE. In particular, hypocomplementemia and antibodies directed against nuclear components; including anti-nuclear antibodies (ANA); extractable nuclear antigens (ENA) inclusive of anti-Ro, anti-La, anti-Sm and anti-RNP; and anti-double stranded DNA (dsDNA) antibodies should prompt the clinician to consider a diagnosis of a connective tissue disease or lupus. In addition, it is important to screen any child with suspected SLE for the presence of aPL (75). A number of small studies have examined for the presence of aPL antibodies in juvenile-onset SLE (JSLE). ACL positivity has been reported to be present in 19–89% of patients with JSLE, whilst LAC has been described in between 10 and 62% of cases (76–79).

The key differences between pediatric onset APS and the adult onset form of the disease are summarized in Table 3.

**Table 3:** Key clinical and seroloe:ical differences between pediatric and adult-onset APS.

**Pediatric onset APS**	**Adult onset APS**
PM related symptoms are rarely seen in the pediatric population in view of the fact that the incidence of pregnancy in this age group is low and limited to the
adolescent population	Disease commonly manifests with PM and *I* or VT related symptoms
No pediatric specific diagnostic criteria available	The updated Sapporo Criteria are a reliable and reproducible diagnostic criteria of high sensitivity and specificity
Often occurs in the context of no other classical pro- thrombotic risk factors	Disease may present in the setting of additional pro- coagulant risk factors including atherosclerosis and hypertension
Typical even incidence between males and females (l: 1.2)	Female predominance (1:5)
Primary APS accounting for between 38-50% of cases	Primary APS accounting for more than half of cases
Most common presenting symptom is VT; predominantly in the lower limb. PM symptoms seldom seen due to the low incidence of pregnancy in
the pediatric group	Patients may present with either VT or PM related symptoms
Antibodies directed against aCL are most commonly
occurring APS antibody (80%)	Antibodies directed against aCL are seen in just over half of adult cases

## Treatment and long-term outcomes

Prompt recognition of the disease and early initiation of treatment is vital so as to reduce the long-term sequelae of VT related symptoms in childhood APS. Residual neurological deficit from cerebral thrombosis may result in a number of development delays in both motor and cognitive function. It is however important to consider that in the majority of cases of pediatric APS the first presentation of the disease is with VT and so there is often little that can be done in terms of primary prevention. In view of the scarcity of cases of pediatric APS, there is little in the way of prospective studies in particular with regards to stratifying treatment of the disorder. Treatment recommendations are therefore usually adapted from evidence based in the literature of adult-onset disease.

The management of pediatric APS however poses a number of importance considerations that are unique to this age group. In view of this the SHARE initiative was launched in order to develop an evidence base for rare rheumatic diseases in childhood including APS.

### Anti-coagulation

The recommendations for treatment were published in 2017 and suggested the use of long-term anticoagulation (most commonly with warfarin) in the case of any child with a positive aPL antibody and venous thrombosis (80). For arterial VT it was also recommended that in addition to long-term anticoagulant therapy consideration for combination therapy with anti-aggregation / anti-platelet agents such as aspirin or clopidogrel should also be considered. In the occurrence of recurrent thrombosis oral anticoagulation should be continued although the target INR should be increased from 2.0–3.0 to 3.0–4.0. Consideration should also be made for alternate therapies in this incidence, for example extended courses of low molecular weight heparin (LMWH) (80).

### Anti-platelet agents in primary prophylaxis

When considering initiating treatment, it is important to consider any additional pro-thrombotic risk (81). In the context of primary thromboprophylaxis against VT (for example in asymptomatic patients with persistently positive aPL antibodies), the use of low-dose aspirin has been recommended. However, in the adult literature, one small placebo-controlled trial of the use of aspirin failed to show any benefit from the treatment after 2 years of follow-up and so the role of aspirin remains debatable (82).

### The role of immunosuppression

In the primary prophylaxis of pediatric secondary APS coexisting with SLE, the SHARE initiative recommended the use of hydroxychloroquine in addition to aspirin (80). Hydroxychloroquine is also thought to offer anti-thrombotic properties(83).

The role for immunosuppressive treatment in the management of primary childhood APS is uncertain. Recent observational studies have found that a number of patients are treated with corticosteroids in addition to other immunosuppressants, however there is no clear evidence to support the routine use in clinical practice. In the context of secondary pediatric APS, a larger group of patients are on concurrent steroid and immunosuppressive treatment although this is typically targeted to the management of CTD symptoms (64).

In the adult literature, rituximab (a chimeric anti-CD20 monoclonal antibody) has been shown to result in significant reduction in IgG aCL antibodies in patients receiving treatment for SLE. However, there are currently no detailed studies of the role of B-cell depletion therapy in either adult or pediatric cases of APS (84).

### Newer oral anti-coagulants

Newer therapeutic targets are now being investigated in adult-onset APS. The RAPS trial recently investigated the use of rivaroxaban (an oral direct factor Xa inhibitor) in comparison to warfarin (the current standard of care in APS) as a treatment of APS in patients with previous venous thromboembolism. The study concluded that there was no increase in thrombosis in patients treated with rivaroxaban in comparison to those taking warfarin (85). The advantage of rivaroxaban is the wider therapeutic window and not monitoring of any markers of coagulation is necessary. However, there are only case reports of the use of rivaroxaban in children with APS and its routine treatment at the point in time of writing this chapter in children with APS is not recommended (86).

Key considerations in the diagnosis and management of pediatric onset APS are summarized in Table 4.

**Table 4:** Key considerations relating to the diagnosis and management of pediatric APS.

•	Lack of PM related symptoms seen in pediatric-onset APS thus rendering widely accepted c lassification for APS unreliable in the childhood cases
•	All children with unproved thromboembolic disease should be screen for aPL antibodies
•	Young patients with JSLE should be routine ly screened for aPL antibodies
•	It is important to consider a variety of widespread systemic symptoms associated with APS
•	Management centers on anticoagulation to reduce the risk of VT
•	Oral anticoagulants (such as warfarin) are the first line treatment although newer agents such as rivaroxaban are currently be assessed for use in adults
•	CAPS accounts for <1% of cases of pediatric APS but can be the presenting symptom of the disease
•	Prompt diagnosis and initiation of treatment with both anticoagulation and immunosuppression is essential in CAPS
•	Essential to ensure both young patients and their families are educated in the treatment of the disease
•	It is vital to check regularly adherence with medication, in particular throughout the adolescent period

### Treatment considerations in pediatric APS

The treatment of APS in childhood is complicated further by a number of key factors that are highly relevant to the management of any young patient with a chronic illness that need to be considered by the treating physician. It is well documented that adherence with treatment in rheumatic diseases amongst adolescent patients is lower than adults. In particular, studies have found that problems with adherence are more likely to be seen in children taking three or more different types of medicine a day (87). In addition to poor adherence with oral anticoagulants, problems occur with LMWH that is given by subcutaneous injection, especially as these injection are often reported to be painful. As this therapy has a shorter half-life than warfarin the implications of missing even occasional doses are especially significant and increase the risk of further VT. The rates of recurrent thrombosis are higher in pediatric cases of APS than in adults although it is not clear as to whether this is in relation to more aggressive disease or as a result of adherence with treatment.

Furthermore, it is essential that the treating clinician considers the wide-ranging implications of anticoagulant therapy which may raise issues regarding education and leisure activities. Patients are discouraged from participating in contact sports and therefore may not be able to fully participate in a variety of leisure activities both in and out of school. Another area that requires lifestyle modification for the young patient with APS relates to the use of alcohol, which can interact with warfarin therapy. It is therefore essential that patient education is maintained throughout the adolescent period.

## Special circumstances

### Catastrophic APS in children

Catastrophic APS (CAPS) represents the most acute and severe manifestation of APS. This is characterized by the rapid development of widespread thrombosis in the micro-circulation of several organs resulting in multi-organ failure. It is associated with high rates of mortality and morbidity, even with rapid diagnosis and optimal treatment. The occurrence of CAPS in the population of children with APS is difficult to quantify and in adult onset disease is believed to account for approximately 1% of all cases with APS (88). Data from the CAPS Registry of more than 400 patients with CAPS has suggested that approximately 10% of cases were seen in those under the age of 18 years (89). When compared with adult onset disease, pediatric CAPS was far more likely to be the presenting symptom of the disease (87% of pediatric cases presented with CAPS in comparison to 45% of adult cases). The risk of mortality from CAPS in pediatric cases was 26%, which is lower than the mortality rate reported in adults (which can be as high as 50%) (89, 90). Registry data also found that pediatric-onset disease is more frequently seen in relation to preceding infection when compared with the adult population (89).

The treatment of APS in pediatric cases is based upon therapies that have been suggested to be of benefit in adult-onset disease. There are no large-scale studies that have assessed treatment of CAPS in either adult or pediatric populations however. Management revolves around a combination of anticoagulation and immunosuppression (90). There is little in the way of consensus regarding the most effective immunosuppressive treatment regime although patients are often treated with a combination of drugs. CAPS registry data found that the vast majority of pediatric cases are treated with corticosteroids (76% of patients). In addition to steroids, pediatric patients are often treated with concomitant plasma exchange, cyclophosphamide and intravenous immunoglobulin (IVIg) therapy (89). The use of cyclophosphamide in pediatric cases must be carefully considered in view of the possible implications for future fertility. Gonadotrophic protection should be considered on a case-by-case basis. In the literature, there is evidence to support the use of B-cell depletion therapy with rituximab in cases of both adults and children with CAPS (91, 92).

### Neonatal APS

The onset of APS in neonatal period is an unusual manifestation of the disease. The infant coagulation system shows a number of key differences when compared with adults and this may contribute to onset of the disease in the neonatal period. In the context of pediatric APS it is important to consider the role of naturally occurring anticoagulants such as Protein C and Protein S, which are seen at a lower levels in the neonatal circulation (93, 94). In addition, neonatal plasminogen levels are typically lower than in adults and platelet aggregation is decreased throughout the first year of life (94). Clinical features including any VT, in particular stroke, should prompt consideration of a diagnosis of neonatal APS. It is often difficult to differentiate between neonatal APS and other hereditary pro-thrombotic disorders and therefore early specialist input is essential in such cases.

### Children born to mothers with APS

Recent studies have focused on the outcomes for children born to mothers with APS. Registry data has observed that aCL antibodies can be detected in newborns suggesting that the antibody is able to cross the placenta. These antibodies typically clear the infant's circulation within the first six months of life. In addition, the occurrence of VT these infants are rare (95). Interestingly, it has also been reported that ß2GPI are seldom able to cross the placenta and contribute little in the way of prothrombotic risk to the newborn (96). In a study of 134 babies born to mothers with APS there were no cases of neonatal thrombosis of SLE (97).

## Conclusions

In conclusion, cases of APS have been described in children of all ages with the disease occasionally manifesting in the neonatal period. The disease is rare in childhood but should be considered in all cases of unprovoked thromboembolic disease. Furthermore, all patients with JSLE should be screened for secondary APS. A number of key differences are seen when comparing the disease in childhood with the more well described adult onset form of the disease. Evidently the rates of PM related symptoms are seldom seen in the childhood form of the disease. Children with APS are also at an increased risk of recurrent thrombotic events when compared with adults and so it is essential to maintain long-term anticoagulant therapy. There is a lack of prospective studies in the field of pediatric APS and so treatment recommendations are largely adapted from adult studies. Pediatric APS represents a leading cause of VT in childhood and can result in potentially devastating long-term complications.

## Author contributions

CW and YI both contributed to the writing of this manuscript. CW was responsible for drafting the manuscript and this was reviewed by YI. Both authors approved the final version.

### Conflict of interest statement

YI is currently an employee of UCB Pharma. The remaining author declare that the research was conducted in the absence of any commercial or financial relationships that could be construed as a potential conflict of interest.

## References

[1] Pericleous C Ripoll VM Giles I Ioannou Y. Laboratory tests for the antiphospholipid syndrome. Methods Mol Biol. (2014) 1134:221–35. 10.1007/978-1-4939-0326-9_17 24497366

[2] Moffat K Raby A Crowther M. Lupus anticoagulant testing. Methods Mol Biol. (2013) 992:97–108. 10.1007/978-1-62703-339-8_7 23546707

[3] Giannakopoulos B Passam F Ioannou Y Krilis SA. How we diagnose the antiphospholipid syndrome. Blood (2009) 113:985–94. 10.1182/blood-2007-12-129627 18755986

[4] Miyakis S Lockshin MD Atsumi T Branch DW Brey RL Cervera R. International consensus statement on an update of the classification criteria for definite antiphospholipid syndrome. J Thromb Haemost. (2006) 4:295–306. 10.1111/j.1538-7836.2006.01753.x 16420554

[5] Win N Islam SI Peterkin MA Walker ID. Positive direct antiglobulin test due to antiphospholipid antibodies in normal healthy blood donors. Vox Sang. (1997) 72:82–4. 10.1159/000461988 9145490

[6] Chandler JB Torres R Rinder HM Tormey CA. Lupus anticoagulant testing and anticoagulation do not mix: quantitation of discrepant results and potential approaches to reduce false positives. Br J Haematol. (2014) 167:704–7. 10.1111/bjh.13030 25041401 PMC7024602

[7] Chighizola CB Gerosa M Trespidi L Di Giacomo A Rossi F Acaia B. Update on the current recommendations and outcomes in pregnant women with antiphospholipid syndrome. Expert Rev Clin Immunol. (2014) 10:505–17. 10.1586/1744666X.2014.968129 25300673

[8] Ruffatti A Salvan E Del Ross T Gerosa M Andreoli L Maina A. Treatment strategies and pregnancy outcomes in antiphospholipid syndrome patients with thrombosis and triple antiphospholipid positivity. A European multicentre retrospective study. Thromb Haemost. (2014) 112:727–35. 10.1160/TH14-03-0191 25008944

[9] Stojanovich L Djokovic A Kontic M. Antiphospholipid-mediated thrombosis: interplay between type of antibodies and localisation of lung, and cardiovascular incidences in primary antiphospholipid syndrome. Clin Exp Rheumatol. (2015) 33:531–6 26088955

[10] Chighizola CB Ubiali T Meroni PL. Treatment of thrombotic antiphospholipid syndrome: the rationale of current management-an insight into future approaches. J Immunol Res. (2015) 2015:951424. 10.1155/2015/951424 26075289 PMC4436516

[11] Ruiz-Irastorza G Crowther M Branch W Khamashta MA. Antiphospholipid syndrome. Lancet (2010) 376:1498–509. 10.1016/S0140-6736(10)60709-X 20822807

[12] Emmi G Silvestri E Squatrito D Ciucciarelli L Cameli AM Denas D. An approach to differential diagnosis of antiphospholipid antibody syndrome and related conditions. Sci World J. (2014) 2014:341342. 10.1155/2014/341342 25374937 PMC4211159

[13] Meroni PL Argolini LM Pontikaki I. What is known about pediatric antiphospholipid syndrome? Expert Rev Hematol. (2016) 9:977–85. 10.1080/17474086.2016.1235969 27615277

[14] Ravelli A Martini A. Antiphospholipid antibody syndrome in pediatric patients. Rheum Dis Clin North Am. (1997) 23:657–76. 10.1016/S0889-857X(05)70351-3 9287381

[15] Chen PP Giles I. Antibodies to serine proteases in the antiphospholipid syndrome. Curr Rheumatol Rep. (2010) 12:45–52. 10.1007/s11926-009-0072-7 20425533 PMC2824841

[16] Meroni PL Raschi E Camera M Testoni C Nicoletti F Tincani A. Endothelial activation by aPL: a potential pathogenetic mechanism for the clinical manifestations of the syndrome. J Autoimmun. (2000) 15:237–40. 10.1006/jaut.2000.0412 10968917

[17] Krone KA Allen KL McCrae KR. Impaired fibrinolysis in the antiphospholipid syndrome. Curr Rheumatol Rep. (2010) 12:53–7. 10.1007/s11926-009-0075-4 20425534 PMC2862601

[18] Nojima J Suehisa E Kuratsune H Machii T Koike T Kitani T. Platelet activation induced by combined effects of anticardiolipin and lupus anticoagulant IgG antibodies in patients with systemic lupus erythematosus–possible association with thrombotic and thrombocytopenic complications. Thromb Haemost. (1999) 81:436–41. 10.1055/s-0037-1614491 10102474

[19] Meroni PL Borghi MO Raschi E Ventura D Sarzi Puttini PC. Inflammatory response and the endothelium. Thromb Res. (2004) 114:329–4. 10.1016/j.thromres.2004.06.045 15507262

[20] Cheng S Wang H Zhou H. The role of TLR4 on B cell activation and Anti-beta2GPI antibody production in the antiphospholipid syndrome. J Immunol Res. (2016) 2016:1719720 10.1155/2016/1719720 27868072 PMC5102736

[21] Laplante P Fuentes R Salem D Subang R Gillis MA Hachem A. Antiphospholipid antibody-mediated effects in an arterial model of thrombosis are dependent on Toll-like receptor 4. Lupus (2016) 25:162–76. 10.1177/0961203315603146 26391610

[22] Avcin T Cimaz R Silverman ED Cervera R Gattorno M Garay S. Pediatric antiphospholipid syndrome: clinical and immunologic features of 121 patients in an international registry. Pediatrics (2008) 122:e1100–7. 10.1542/peds.2008-1209 18955411

[23] Miesbach W. Antiphospholipid antibodies and antiphospholipid syndrome in patients with malignancies: features, incidence, identification, and treatment. Semin Thromb Hemost. (2008) 34:282–5. 10.1055/s-0028-1082272 18720308

[24] De Wolf F Carreras LO Moerman P Vermylen J Van Asscher A Renaer M. Decidual vasculopathy and extensive placental infarction in a patient with repeated thromboembolic accidents, recurrent fetal loss, and a lupus anticoagulant. Am J Obstet Gynecol. (1982) 142:829–34. 10.1016/S0002-9378(16)32527-3 6801984

[25] Hanly JG Gladmann DD Rose TH Laskin CA Urowitz MB. Lupus pregnancy. A prospective study of placental changes. Arthritis Rheum. (1988) 31:358–66. 10.1002/art.1780310307 3128986

[26] Nayar R Lage JM. Placental changes in a first trimester missed abortion in maternal systemic lupus erythematosus with antiphospholipid syndrome; a case report and review of the literature. Hum Pathol. (1996) 27:201–6. 10.1016/S0046-8177(96)90377-9 8617465

[27] Willis R Harris EN Pierangeli SS. Pathogenesis of the antiphospholipid syndrome. Semin Thromb Hemost. (2012) 38:305–21. 10.1055/s-0032-1311827 22510982

[28] de Groot PG Urbanus RT Antiphospholipid syndrome - not a noninflammatory disease. Semin Thromb Hemost. (2015) 41:607–14. 10.1055/s-0035-1556725 26276935

[29] Gharavi AE Pierangeli SS Espinola RG Liu X Colden-Stanfield M Harris EN. Antiphospholipid antibodies induced in mice by immunization with a cytomegalovirus-derived peptide cause thrombosis and activation of endothelial cells in vivo. Arthritis Rheum (2002) 46:545–52. 10.1002/art.10130 11840458

[30] Dagenais P Urowitz MB Gladman DD Norman CS. A family study of the antiphospholipid syndrome associated with other autoimmune diseases. J Rheumatol. (1992) 19:1393–6. 1433007

[31] Asherson RA Doherty DG Verganim D Khamashta MA Hughes GR. Major histocompatibility complex associations with primary antiphospholipid syndrome. Arthritis Rheum. (1992) 35:124–5. 10.1002/art.1780350119 1731810

[32] Sebastiani GD Iuliano A Cantarini L Galeazzi M. Genetic aspects of the antiphospholipid syndrome: An update. Autoimmun Rev. (2016) 15:433–9. 10.1016/j.autrev.2016.01.005 26804759

[33] Papalardo E Romay-Penabad Z Willis R Christadoss P Carrera-Marin AL Reyes-Maldonado E. Major histocompatibility complex class II alleles influence induction of pathogenic antiphospholipid antibodies in a mouse model of thrombosis. Arthritis Rheumatol. (2017) 69:2052–61. 10.1002/art.40195 28666081

[34] Fredi M Tincani A Yin H Delgado-Vega AM Borghi MO Meroni PL IRF5 is associated with primary antiphospholipid syndrome, but is not a major risk factor. Arthritis Rheumatism. (2010) 62:1201–2. 10.1002/art.27345 20131273

[35] Yin H Borghi MO Delgardo-Vega AM Tincani A Meroni PL Alarcón-Riquelme ME Association of STAT4 and BLK, but not BANK1 or IRF5, with primary antiphospholipid syndrome. Arthritis Rheum. (2009) 60:2468–71. 10.1002/art.24701 19644876

[36] Berkun Y Padeh S Barash J Uziel Y Harel L Mukamel M. Antiphospholipid syndrome and recurrent thrombosis in children. Arthritis Rheum. (2006) 55:850–5. 10.1002/art.22360 17139660

[37] Cervera R Piette JC Font J Khamashta MA Shoenfeld Y Camps MT. Antiphospholipid syndrome: clinical and immunologic manifestations and patterns of disease expression in a cohort of 1,000 patients. Arthritis Rheum. (2002) 46:1019–27. 10.1002/art.10187 11953980

[38] Ambrose N Morgan TA Galloway J Ionnaou Y Beresford MW Isenberg DA. Differences in disease phenotype and severity in SLE across age groups. Lupus (2016) 25:1542–50. 10.1177/0961203316644333 27147622 PMC5089221

[39] Aguiar CL Soybilgic A Avcin T Myones BL. Pediatric Antiphospholipid Syndrome. Curr Rheumatol Rep. (2015) 17:27. 10.1007/s11926-015-0504-5 25854492

[40] Andrews A Hickling P. Thrombosis associated with antiphospholipid antibody in juvenile chronic arthritis. Lupus (1997) 6:556–7. 10.1177/096120339700600616 9256318

[41] Monastiri K Selmi H Tabarki B Yacoub M Mahjoub T Essoussi AS. Primary antiphospholipid syndrome presenting as complicated Henoch-Schonlein purpura. Arch Dis Child. (2002) 86:132–3. 10.1136/adc.86.2.132 11827910 PMC1761071

[42] Meglič A Grosek S Benedik-Dolnicar M Avcin T. Atypical haemolytic uremic syndrome complicated by microangiopathic antiphospholipid-associated syndrome. Lupus (2008) 17:842–5. 10.1177/0961203308091634 18755867

[43] Sherer Y Livneh A Levy Y Shoenfeld Y Langevitz P. Dermatomyositis and polymyositis associated with the antiphospholipid syndrome-a novel overlap syndrome. Lupus (2000) 9:42–6. 10.1177/096120330000900108 10713646

[44] Kiechl-Kohlendorfer U Ellemunter H Kiechl S. Chorea as the presenting clinical feature of primary antiphospholipid syndrome in childhood. Neuropediatrics (1999) 30:96–8. 10.1055/s-2007-973468 10401693

[45] Cervera R Serrano R Pons-Estel GJ Ceberio-Hualde L Shoenfeld Y de Ramón E. Morbidity and mortality in the antiphospholipid syndrome during a 10-year period: a multicentre prospective study of 1000 patients. Ann Rheum Dis. (2015) 74:1011–8. 10.1136/annrheumdis-2013-204838 24464962

[46] Gattorno M Falcini F Ravelli A Zulian F Buoncompagni A Martini G. Outcome of primary antiphospholipid syndrome in childhood. Lupus (2003) 12:449–53. 10.1191/0961203303lu411oa 12873046

[47] Gomez-Puerta JA Martín H Amigo MC Aguirre MA Camps MT Cuadrado MJ. Long-term follow-up in 128 patients with primary antiphospholipid syndrome: do they develop lupus? Medicine (2005) 84:225–30. 10.1097/01.md.0000172074.53583.ea 16010207

[48] Avcin T de Graeff N Bader-Meunier B Brogan P Dolezalova P Feldman B Evidence based recommendations for diagnosis and treatment of the antiphospholipid syndrome in children - results of the share initiative. Lupus (2016) 25(Suppl. 1):4–100. 10.1136/annrheumdis-2016-210960

[49] von Scheven E Athreya BH Rose CD Goldsmith DP Morton L. Clinical characteristics of antiphospholipid antibody syndrome in children. J Pediatr. (1996) 129:339–45. 10.1016/S0022-3476(96)70064-1 8804321

[50] Di Nucci GD Mariani G Arcieri P Cerbo R Tarani L Bruni L. Antiphospholipid syndrome in young patients. Two cases of cerebral ischaemic accidents Eur J Pediatr. (1995) 154:334. 10.1007/BF01957376 7607290

[51] Crowther MA Ginsberg JS Julian J Denburg J Hirsh J Douketis J. A comparison of two intensities of warfarin for the prevention of recurrent thrombosis in patients with the antiphospholipid antibody syndrome. N Engl J Med. (2003) 349:1133–8. 10.1056/NEJMoa035241 13679527

[52] Berkun Y Simchen MJ Strauss T Menaschu S Padeh S Kenet G Antiphospholipid antibodies in neonates with stroke–a unique entity or variant of antiphospholipid syndrome? Lupus (2014) 23:986–93. 10.1177/0961203314531842 24729280

[53] Spalice A Del Balzo F Perla FM Papetti L Nicita F Ursitti F. Pediatric cerebellar stroke associated with elevated titer of antibodies to beta2-glycoprotein. Med Hypotheses. (2011) 76:831–3. 10.1016/j.mehy.2011.02.030 21388749

[54] Avcin T Markelj G Niksic V Rener-Primec Z Cucnik S Zupancic M. Estimation of antiphospholipid antibodies in a prospective longitudinal study of children with migraine. Cephalalgia (2004) 24:831–7. 10.1111/j.1468-2982.2004.00752.x 15377313

[55] Shaharao V Bartakke S Muranjan MN Bavdekar MS Bavdekar SB Udani VP. Recurrent acute transverse myelopathy: association with antiphospholipid antibody syndrome. Indian J Pediatr. (2004) 71:559–61. 10.1007/BF02724305 15226572

[56] Okun MS Jummani RR Carney PR. Antiphospholipid- associated recurrent chorea and ballism in a child with cerebral palsy. Pediatr Neurol. (2000) 23:62–3. 10.1016/S0887-8994(00)00152-1 10963973

[57] Campos LM Kiss MH D'Amico EA Silva CA. Antiphospholipid antibodies and antiphospholipid syndrome in 57 children and adolescents with systemic lupus erythematosus. Lupus (2003) 12:820–6. 10.1191/0961203303lu471oa 14667097

[58] Suvajac G Stojanovich L Milenkovich S. Ocular manifestations in antiphospholipid syndrome. Autoimmun Rev. (2007) 6:409–14. 10.1016/j.autrev.2006.11.005 17537387

[59] Golstein M Meyer O Bourgeois P Palazzo E Nicaise P Labarre C. Neurological manifestations of systemic lupus erythematosus: role of antiphospholipid antibodies. Clin Exp Rheumatol. (1993) 11:373–9. 8403581

[60] Miller DJ Maisch SA Perez MD Kearney DL Feltes TF. Fatal myocardial infarction in an 8-year-old girl with systemic lupus erythematosus, Raynaud's phenomenon, and secondary antiphospholipid antibody syndrome. J Rheumatol. (1995) 22:768–73. 7791180

[61] Moreno-Ruiz LA Mendoza-Pérez BC Santillano-Gómez E Jiménez-Arteaga S Borrayo-Sánchez G Alva-Espinosa C. Non-ST-elevation acute myocardial infarction secondary to antiphospholipid antibody syndrome in an adolescent female. Cir Cir. (2010) 78:435–8 21219815

[62] Clauss SB Manco-Johnson MJ Qiovers E Takemoto C Spevak PJ. Primary antiphospholipid antibody syndrome and cardiac involvement in a child. Pediatr Cardiol. (2003) 24:292–4. 10.1007/s00246-002-0273-6 12632226

[63] Bhat MA Qureshi UA Ali SW Bhat JI Robbani I. Pulmonary thromboembolism as the initial manifestation in a child with antiphospholipid syndrome in the emergency department. Pediatr Emerg Care (2011) 27:205–7. 10.1097/PEC.0b013e31820d8dd4 21378521

[64] Ma J Song H Wei M He Y. Clinical characteristics and thrombosis outcomes of paediatric antiphospholipid syndrome: analysis of 58 patients. Clin Rheumatol. (2018) 37:1295–303. 10.1007/s10067-017-3776-5 28748509

[65] Noda S Ogura M Tsutsumi A Udagawa T Kamei K Matsuoka K. Thrombotic microangiopathy due to multiple autoantibodies related to antiphospholipid syndrome. Pediatr Nephrol. (2012) 27:681–5. 10.1007/s00467-011-2085-5 22210384

[66] Butani L. End-stage renal disease from glomerulonephritis associated with anti-phospholipid syndrome. Pediatr Nephrol. (2004) 19:812–4. 10.1007/s00467-004-1491-3 15133727

[67] Minisola G Porzio V Bancheri C Ceralli F Carnabuci A Onetti Muda A. Atypical renal onset and involvement in primary antiphospholipid syndrome in a child. Clin Exp Rheumatol. (1998) 16:102–4 9543576

[68] Ramon I Mathian A Bachelot A Hervier B Haroche J Boutin-Le Thi Huong D. Primary adrenal insufficiency due to bilateral adrenal hemorrhage-adrenal infarction in the antiphospholipid syndrome: long-term outcome of 16 patients. J Clin Endocrinol Metab. (2013) 98:3179–89. 10.1210/jc.2012-4300 23783099

[69] Tavil B Ozyurek E Gumruk F Cetin M Gurgey A. Antiphospholipid antibodies in Turkish children with thrombosis. Blood Coagul Fibrinolysis. (2007) 18:347–52. 10.1097/MBC.0b013e32809cc95a 17473576

[70] Chang AD Tachdjian R Gallagher K McCurdy DK Lassman C Stiehm ER. Type III mixed cryoglobulinemia and antiphospholipid syndrome in a patient with partial DiGeorge syndrome. Clin Dev Immunol. (2006) 13:261–4. 10.1080/17402520600877778 17162366 PMC2270767

[71] Hunt BJ. Pediatric antiphospholipid antibodies and antiphospholipid syndrome. Semin Thromb Hemost. (2008) 34:274–81. 10.1055/s-0028-1082271 18720307

[72] Avčin T Ambrozic A Kuhar M Kveder T Rozman B Anticardiolipin and anti-β2 glycoprotein I antibodies in sera of 61 apparently healthy children at regular preventive visits. Rheumatology (2001) 40:565–73. 10.1093/rheumatology/40.5.565 11371668

[73] Pericleous C Ferreira I Borghi O Pregnolato F McDonnell T Garza-Garcia A. Measuring IgA Anti-beta2-Glycoprotein I, and IgG/IgA Anti-Domain I antibodies adds value to current serological assays for the antiphospholipid syndrome. PLoS ONE (2016) 11:e0156407. 10.1371/journal.pone.0156407 27253369 PMC4890741

[74] Cousins L Pericleous C Khamashta M Bertolaccini ML Ioannou Y Giles I, Antibodies to domain I of beta-2-glycoprotein I and IgA antiphospholipid antibodies in patients with 'seronegative' antiphospholipid syndrome. Ann Rheum Dis. (2015) 74:317–9. 10.1136/annrheumdis-2014-206483 25359383 PMC4283613

[75] Avcin T Silverman ED. Antiphospholipid antibodies in pediatric systemic lupus erythematosus and the antiphospholipid syndrome. Lupus (2007) 16:627–33. 10.1177/0961203307079036 17711899

[76] Montes de Oca MA Babron MC Blétry O Broyer M Courtecuisse V Fontaine JL Thrombosis in systemic lupus erythematosus: a French collaborative study. Arch Dis Child. (1991) 66:713–7. 10.1136/adc.66.6.713 1905123 PMC1793157

[77] Molta C Meyer O Dosquet C Montes de Oca M Babron MC Danon F. Childhood-onset systemic lupus erythematosus: antiphospholipid antibodies in 37 patients and their first-degree relatives. Pediatrics (1993) 92:849–53 8233748

[78] Massengill SF Hedrick C Ayoub EM Sleasman JW Kao KJ. Antiphospholipid antibodies in pediatric lupus nephritis. Am J Kidney Dis. (1997) 29:355–61. 10.1016/S0272-6386(97)90195-5 9041210

[79] Seaman DE Londino AV Kwoh CK Medsger TA Manzi S. Antiphospholipid antibodies in pediatric systemic lupus erythematosus. Pediatrics (1995) 96:1040–5. 7491218

[80] Groot N de Graeff N Avcin T Bader-Meunier B Dolezalova P Feldman B. European evidence-based recommendations for diagnosis and treatment of paediatric antiphospholipid syndrome: the SHARE initiative. Ann Rheum Dis. (2017) 76:1637–41. 10.1136/annrheumdis-2016-211001 28473426

[81] Ruiz-Irastorza G Cuadrado MJ Ruiz-Arruza I Brey R Crowther M Deerksen R. Evidence-based recommendations for the prevention and long-term management of thrombosis in antiphospholipid antibody-positive patients: report of a task force at the 13th International Congress on antiphospholipid antibodies. Lupus (2011) 20:206–18. 10.1177/0961203310395803 21303837

[82] Erkan D Harrison ML Levy R Peterson M Petri M Sammaritano L. Aspirin for primary thrombosis prevention in the antiphospholipid syndrome: a randomized, double-blind, placebo-controlled trial in asymptomatic antiphospholipid antibody-positive individuals. Arthritis Rheum. (2007) 56:2382–91. 10.1002/art.22663 17599766

[83] Belizna C. Hydroxychloroquine as an anti-thrombotic in antiphospholipid syndrome. Autoimmun Rev. (2015) 14:358–62. 10.1016/j.autrev.2014.12.006 25534016

[84] Ioannou Y Lambrianides A Cambridge G Leandro MJ Edwards JC Isenberg DA. B cell depletion therapy for patients with systemic lupus erythematosus results in a significant drop in anticardiolipin antibody titres. Ann Rheum Dis. (2008) 67:425–6. 10.1136/ard.2007.078402 17905784

[85] Cohen H Hunt BJ Efthymiou M Arachchillage DR Mackie IJ Clawson S. Rivaroxaban versus warfarin to treat patients with thrombotic antiphospholipid syndrome, with or without systemic lupus erythematosus (RAPS): a randomised, controlled, open-label, phase 2/3, non-inferiority trial. Lancet Haematol. (2016) 3:e426–36. 10.1016/S2352-3026(16)30079-5 27570089 PMC5010562

[86] Ma JY Zhang X Li XF He LJ Ma N Wei YY. Thrombotic storm in a 4-year-old boy with a thrombus in the right atrium. Int J Immunopathol Pharmacol. (2018) 32:2058738418778121. 10.1177/2058738418778121 29798687 PMC5971376

[87] Bugni VM Ozaki L Okamoto KY Barbosa CM Hilário MO Len CA. Factors associated with adherence to treatment in children and adolescents with chronic rheumatic diseases. J Pediatr. (2012) 88:483–8. 10.2223/JPED.2227 23269234

[88] Asherson RA. The catastrophic antiphospholipid syndrome. J Rheumatol. (1992) 19:508–12 1593568

[89] Berman H Rodríguez-Pintó I Cervera R Gregory S ds Meis E Rodriquies CE. Pediatric catastrophic antiphospholipid syndrome: descriptive analysis of 45 patients from the ”CAPS Registry“. Autoimmun Rev. (2014) 13:157–62. 10.1016/j.autrev.2013.10.004 24145009

[90] Asherson RA Cervera R Piette JC Font J Lie JT Burcoglu A. Catastrophic antiphospholipid syndrome. Clinical and laboratory features of 50 patients. Medicine (1998) 77:195–207. 10.1097/00005792-199805000-00005 9653431

[91] Iglesias-Jiménez E Camacho-Lovillo M Falcón-Neyra D Lirola-Cruz J Neth O. Infant with probable catastrophic antiphospholipid syndrome successfully managed with rituximab. Pediatrics (2010) 125:e1523–8. 10.1542/peds.2009-2939 20478943

[92] Berman H Rodriguez-Pintó I Cervera R Morel N Costedoat-Chalumeau N Erkan D. Rituximab use in the catastrophic antiphospholipid syndrome: descriptive analysis of the CAPS registry patients receiving rituximab. Autoimmun Rev. (2013) 12:1085–90. 10.1016/j.autrev.2013.05.004 23777822

[93] Thornburg C Pipe S. Neonatal thromboembolic emergencies. Semin Fetal Neonatal Med. (2006) 11:198–206. 10.1016/j.siny.2006.01.005 16520103

[94] Nowak-Göttl U Kurnik K Manner D Kenet G. Thrombophilia testing in neonates and infants with thrombosis. Semin Fetal Neonatal Med. (2011) 16:345–8. 10.1016/j.siny.2011.07.005 21835708

[95] Boffa MC Lachassinne E. Infant perinatal thrombosis and antiphospholipid antibodies: a review. Lupus (2007) 16:634–41. 10.1177/0961203307079039 17711900

[96] Meroni PL Borghi MO Raschi E Tedesco F. Pathogenesis of antiphospholipid syndrome: understanding the antibodies. Nat Rev Rheumatol. (2011) 7:330–9. 10.1038/nrrheum.2011.52 21556027

[97] Mekinian A Lachassinne E Nicaise-Roland P Carbillon L Motta M Vicaut E. European registry of babies born to mothers with antiphospholipid syndrome. Ann Rheum Dis. (2013) 72:217–22. 10.1136/annrheumdis-2011-201167 22589374 PMC3551221

